# Alignment of national COVID-19 vaccine recommendations for pregnant and lactating women

**DOI:** 10.2471/BLT.21.286644

**Published:** 2021-08-30

**Authors:** Michelle L Giles, Ahinsa Gunatilaka, Kirsten Palmer, Ketaki Sharma, Vijay Roach

**Affiliations:** a Department of Obstetrics and Gynaecology, Monash University, 246 Clayton Road, Clayton, Victoria 3168, Melbourne, Australia.; b National Centre for Immunisation Research and Surveillance, Sydney, Australia.; c Royal Australian and New Zealand College of Obstetricians and Gynaecologists, Melbourne, Australia.

## Abstract

The rapid development and roll-out of coronavirus disease 2019 (COVID-19) vaccines is providing hope for a way to control the pandemic. As pregnant and lactating women are generally excluded from clinical trials, the vaccination programme was launched without adequate safety and efficacy data for pregnant women. Yet many professional organizations have recognized the need for administration of COVID-19 vaccines in pregnancy and have issued their own set of recommendations. The lack of evidence, however, has often led to confused messaging, inconsistent language and differing recommendations across organizations, potentially contributing to delay or refusal to accept vaccination by pregnant women. We summarize those differences and recommend that leaders collaborate at a country level to produce joint recommendations. We use the example of Australia, where two professional authorities along with the government and partners in New Zealand worked towards one message, consistent language and a unified recommendation. The aim was to help health professionals and women who are planning pregnancy or who are currently pregnant or breastfeeding to make an informed decision about COVID-19 vaccination. National advisory groups for immunization, professional obstetric organizations and government bodies should be encouraged to coordinate their statements on COVID-19 vaccination for pregnant and lactating women and to use similar language and phrasing for greater clarity.

## Résumé

La rapidité de développement et de déploiement des vaccins contre la maladie à coronavirus 2019 (COVID-19) entretient l'espoir d'un jour pouvoir contrôler la pandémie. Étant donné que les femmes enceintes et allaitantes sont généralement exclues des essais cliniques, le programme de vaccination a été lancé en l'absence de données adéquates sur l'efficacité et l'innocuité du vaccin au sein de cette catégorie. Pourtant, de nombreuses associations professionnelles ont reconnu la nécessité de vacciner contre la COVID-19 durant la grossesse, et ont émis leurs propres recommandations. L'absence de preuves a toutefois souvent donné lieu à une communication incohérente, à un discours contradictoire et à des recommandations divergentes d'une organisation à l'autre, ce qui pourrait avoir conduit certaines femmes enceintes à retarder ou refuser la vaccination. Dans le présent document, nous exposons ces différences et exhortons les dirigeants à collaborer au niveau national pour formuler des recommandations communes. Nous utilisons l'exemple de l'Australie, où deux autorités professionnelles ont travaillé avec le gouvernement et des partenaires en Nouvelle-Zélande afin de transmettre un message unique, d'adopter un langage cohérent et de fournir des directives homogènes. L'objectif était d'aider les soignants et les femmes enceintes, allaitantes ou planifiant une grossesse à prendre une décision éclairée en matière de vaccination contre la COVID-19. Les organes consultatifs nationaux sur la vaccination, les associations professionnelles d'obstétrique et les pouvoirs publics devraient être encouragés à aligner leurs déclarations concernant la vaccination contre la COVID-19 chez les femmes enceintes et allaitantes, ainsi qu'à employer des termes et énoncés similaires pour davantage de clarté.

## Resumen

El rápido desarrollo y puesta en marcha de las vacunas contra el coronavirus de la enfermedad por coronavirus (COVID-19) está dando esperanzas sobre una forma de controlar la pandemia. Como las mujeres embarazadas y lactantes suelen estar excluidas de los ensayos clínicos, el programa de vacunación se puso en marcha sin datos adecuados de seguridad y eficacia para las mujeres embarazadas. Sin embargo, muchas organizaciones profesionales han reconocido la necesidad de administrar las vacunas contra la COVID-19 durante el embarazo y han emitido su propio conjunto de recomendaciones. Sin embargo, la falta de pruebas a menudo ha ocasionado mensajes confusos, un lenguaje incoherente y recomendaciones diferentes en las distintas organizaciones, lo que puede contribuir a retrasar o rechazar la vacunación de las mujeres embarazadas. Resumimos esas diferencias y recomendamos que los líderes colaboren a nivel de país para elaborar recomendaciones conjuntas. Utilizamos el ejemplo de Australia, donde dos autoridades profesionales, junto con el gobierno y los socios de Nueva Zelanda, trabajaron para lograr transmitir un único mensaje con un lenguaje coherente y una recomendación unificada. El objetivo era ayudar a los profesionales sanitarios y a las mujeres que planean un embarazo o embarazadas o en periodo de lactancia a tomar una decisión informada sobre la vacunación contra la COVID-19. Hay que animar a los grupos consultivos nacionales de inmunización, a las organizaciones profesionales de obstetricia y a los organismos gubernamentales a que coordinen sus declaraciones sobre la vacunación contra la COVID-19 para las mujeres embarazadas y lactantes y a que utilicen un lenguaje y una redacción similares para mayor claridad.

## ملخص

إن التطور والطرح السريع للقاحات مرض فيروس كورونا 2019 (كوفيد 19)، يمنحان الأمل في توفير طريقة للسيطرة على الجائحة. ونظرًا لأن النساء الحوامل والمرضعات يتم استبعادهن عمومًا من التجارب الإكلينيكية، فقد تم إطلاق برنامج التطعيم دون توفر بيانات كافية عن السلامة والفعالية بالنسبة للحوامل. إلا أن العديد من المنظمات المهنية قد أدركت الحاجة إلى إعطاء لقاحات كوفيد 19 أثناء الحمل، وأصدرت مجموعة من التوصيات الخاصة بها. ومع ذلك، فقد أدى الافتقار لوجود أدلة في كثير من الأحيان إلى رسائل مربكة، ولغة غير متناغمة، وتوصيات مختلفة من المنظمات المختلفة، وهو ما قد يساهم في تأخير أو رفض قبول التطعيم من جانب النساء الحوامل. نحن نلخص هذه الاختلافات، ونوصي بأن يقوم القادة بالتعاون على مستوى الدولة لوضع توصيات مشتركة. نحن نستخدم المثال الخاص بأستراليا، حيث عملت سلطتان مهنيتان، جنبًا إلى جنب، مع الحكومة، والشركاء في نيوزيلندا معًا، من أجل صياغة رسالة واحدة، ولغة متناغمة، وتوصية موحدة. كان الهدف هو مساعدة أخصائيي الصحة والنساء اللواتي يخططن للحمل، أو الحوامل أو المرضعات حاليًا، لاتخاذ قرار مستنير بشأن تطعيم كوفيد 19. يجب تشجيع المجموعات الاستشارية الوطنية للتحصين، ومنظمات التوليد المهنية، والهيئات الحكومية على تنسيق بياناتها بشأن تطعيم كوفيد 19 للنساء الحوامل والمرضعات، واستخدام لغة وصياغة مماثلة لتحقيق مزيد من الوضوح.

## 摘要

新型冠状病毒肺炎疫苗的快速研发和推广为疫情防控工作带来了希望。由于临床试验通常不纳入孕期和哺乳期妇女,所以疫苗接种计划的推行并没有充足的针对孕期妇女的安全和疗效数据。不过,众多专业组织已经意识到在孕期接种新冠疫苗的必要性,并且发布了自己的一套推荐程序。然而,证据的缺乏往往导致不同组织之间的信息混乱、语言不一致和意见分歧,从而有可能导致孕妇推迟或拒绝接种疫苗。我们总结了这些差异并建议各个组织的领导能够在国家层面开展合作以提出意见统一的推荐程序。我们以澳大利亚为例,该国两个专业的权威机构与政府和新西兰的合作伙伴联合提出以一致的语言,传递统一的讯息和推荐程序。其目的在于帮助医护工作者和打算怀孕或者正处于孕期或哺乳期的妇女针对新冠疫苗接种作出明智的决策。我们应鼓励国家免疫咨询小组、专业的产科机构和政府单位统一他们针对孕期和哺乳期妇女接种新冠疫苗方面的陈述,并使用类似的语言和措辞以便更加明确地阐述其意见。

## Резюме

Быстрая разработка и внедрение вакцин против коронавирусной инфекции 2019 года (COVID-19) вселяет надежду в борьбе с этой пандемией. Поскольку беременные и кормящие женщины обычно не участвуют в клинических испытаниях, программа вакцинации была запущена без надлежащих данных о безопасности и эффективности для беременных женщин. Тем не менее многие профессиональные организации признали необходимость применения вакцины против COVID-19 во время беременности и выпустили свой собственный набор рекомендаций. Однако отсутствие доказательств часто приводило к путанице в информационных сообщениях, противоречащим формулировкам и несогласованным рекомендациям в разных организациях, что потенциально способствовало задержке вакцинации или отказу беременных женщин от вакцинации. Авторы приводят сводку этих различий и рекомендуют руководителям сотрудничать на уровне страны для подготовки совместных рекомендаций. В качестве примера использован опыт Австралии, где два профессиональных органа вместе с правительством и партнерами в Новой Зеландии работали над согласованным информированием, едиными формулировками и рекомендациями. Цель заключалась в том, чтобы помочь медицинским работникам и женщинам, планирующим беременность, беременным или кормящим грудью, принять обоснованное решение по поводу вакцинации против COVID-19. Следует поощрять национальные консультативные группы по иммунизации, профессиональные акушерские организации и государственные органы согласовывать свои заявления о вакцинации против COVID-19 для беременных и кормящих женщин, а также использовать схожие формулировки и выражения для обеспечения большей ясности.

## Introduction

Since the first reported case of coronavirus disease 2019 (COVID-19), there have been more than 201 million people infected globally, leading to more than 4.27 million deaths up to early August 2021.^1^ The development and licensing of COVID-19 vaccines has progressed rapidly, with over 4 billion doses delivered globally.^2^ However, as pregnant and lactating women are generally excluded from clinical trials, the registration and widespread roll-out of COVID-19 vaccines was undertaken without adequate safety and efficacy data for these women.

The protection offered by the vaccine may be especially important as pregnant women are at risk of more severe disease from COVID-19.^3^ They are more likely to be admitted to an intensive care unit, to require mechanical ventilation and to deliver preterm.^3^ Adverse outcomes are more likely in pregnant women, with risk factors for severe disease including older age (older than 35 years), higher body mass index, and comorbidities such as diabetes or hypertension.^3^ Furthermore, health-care workers – the majority of whom are women of reproductive age – have been disproportionately affected in many countries worldwide due to high risk of exposure to severe acute respiratory syndrome coronavirus 2 (SARS-CoV-2).^4^^,^^5^

Keeping up with the evolving evidence and changing guidelines during the COVID-19 pandemic is challenging not only for the general population but also for health professionals. In this article we discuss how inconsistencies in recommendations on COVID-19 vaccination published by expert advisory groups may contribute to patients delaying or refusing to accept vaccination. We propose an approach that brings together national expert advisory groups to reach a national consensus in recommendations for COVID-19 vaccination in pregnant and lactating women. 

## Variation in recommendations

Although clinical trial data are lacking, many professional organizations have recognized the need for the administration of COVID-19 vaccines in pregnancy and have issued recommendations.^6^^–^^9^ Although the messages are broadly similar, there remain some inconsistencies, particularly regarding prioritization of pregnant women,^10^^,^^11^ the recommended timing of vaccination during pregnancy (such as avoidance of vaccination in the first 12 weeks)^12^ and the inclusion of a brand preference for vaccines (for example, based on messenger ribonucleic acid, mRNA, technology rather than viral-vector technology).

In addition to inconsistencies in recommendations, there is often variable language used to convey these messages, combined with a lack of positive messaging about the benefits of vaccination (Table 1). Inconsistent communication, particularly during a pandemic, can cost lives.^20^ Two national polls conducted in the United States of America (USA) revealed that positive and personal language is most likely to motivate people to follow public health recommendations and feel more confident about receiving a COVID-19 vaccine.^21^^,^^22^ What the authors describe as “the language of vaccine acceptance” includes: tailoring the message for the audience; explaining the benefits of getting vaccinated; talking about the medical experts involved in the vaccine development; and avoiding judgemental language. This poll also revealed that people were more likely to trust medical experts than pharmaceutical companies and to have confidence and acceptance with the use of positive language emphasizing the benefits of getting vaccinated.^21^^,^^22^

**Table 1:** Summary of recommendations from peak medical organizations, government and immunization technical advisory groups about COVID-19 vaccination for pregnant and lactating women

Society or organization	Specific patient populations		
	Planning pregnancy	Pregnant	Breastfeeding
Centers for Disease Control and Prevention^13^	“…can receive a COVID-19 vaccine” “…no evidence that… COVID-19 vaccines cause female or male fertility problems” “…does not recommend routine pregnancy testing” “…do not need to avoid pregnancy after”	“If you are pregnant, you can receive a COVID-19 vaccine” “…these vaccines... are unlikely to pose a risk for people who are pregnant” “…early data… did not identify any safety concerns for pregnant people who were vaccinated or their babies” “COVID-19 vaccines and other vaccines may now be administered without regard to timing”	“…lactating people can receive a COVID-19 vaccine” “COVID-19 vaccines are thought not to be a risk to lactating people or their breastfeeding babies”
American College of Obstetricians and Gynecologists^9^	“…claims linking COVID-19 vaccines to infertility are unfounded and have no scientific evidence supporting them” “…recommends vaccination for all eligible people who may consider future pregnancy”	“…recommends that pregnant individuals have access to COVID-19 vaccines”	“COVID-19 vaccines should be offered to lactating individuals similar to non-lactating individuals” “There is no need to avoid initiation or discontinue breastfeeding”
European Society of Human Reproduction and Embryology^14^	“…lack of information on the possible effect… on assisted reproduction treatment or future pregnancy” “…prudent to postpone the start of assisted reproduction”	“…should be informed about the lack of long-term human studies… but should not be excluded from vaccination programmes.” Decision should be made “after considering the benefits and risks”	NR
World Health Organization^15^	“WHO does not recommend pregnancy testing prior to vaccination.” “WHO does not recommend delaying pregnancy”	“…may receive the vaccine if the benefit of vaccinating a pregnant woman outweighs the potential vaccine risks.” “…pregnant women at high risk… may be vaccinated”	“Vaccination can be offered to breastfeeding women if they are part of a group prioritized for vaccination” “WHO does not recommend discontinuation of breastfeeding after vaccination”
Royal College of Obstetricians and Gynaecologists^8^	“…do not need to avoid pregnancy after vaccination” “…no evidence to suggest that COVID-19 vaccines will affect fertility”	“…benefits and risks… should be discussed on an individual basis” “…while there is no known risk associated with giving other non-live vaccines to pregnant women, there are no specific data as yet about the safety of COVID-19 vaccination in pregnancy”	“You should not stop breastfeeding in order to be vaccinated against COVID-19”
Australian Technical Advisory Group on Immunisation^16^	“You do not need to avoid becoming pregnant before or after vaccination. You are not required to have a pregnancy test before getting vaccinated”	“…do not routinely recommend COVID-19 vaccine in pregnancy” “…can consider it if the potential benefits of vaccination outweigh any potential risks”	“…can receive the COVID vaccine at any time, and do not need to stop breastfeeding after vaccination”
Royal Australian and New Zealand College of Obstetricians and Gynaecologists^7^	“…no evidence that women who become pregnant after receiving the vaccine are at increased risk of teratogenicity, miscarriage or maternal illness. Pregnancy need not be delayed after receiving the vaccine”	“While Royal Australian and New Zealand College of Obstetricians and Gynaecologists does not recommend routine universal vaccination in the setting of low community transmission, all pregnant women should receive information” “Women with risk factors advised to consider vaccination”	“…no data on the safety of COVID-19 vaccines in lactating women or on the effects of inactivated vector-based vaccines or mRNA vaccines on the breastfed infant or on milk production/excretion. These vaccines are not thought to be a risk to the breastfeeding infant”
Immunisation Advisory Centre, New Zealand^17^	“Routine pregnancy testing before COVID-19 vaccination is not recommended and those who are trying to become pregnant do not need to avoid pregnancy after receiving a COVID vaccine”	“Women who are pregnant and at risk of exposure to SARS-CoV-2 virus can receive a COVID-19 vaccine at any stage of pregnancy. For those at low risk of exposure, it is recommended to delay vaccination until after birth”	“Breastfeeding women can receive a COVID-19 vaccine. There are no safety concerns for a breastfeeding woman or her infant associated with having this COVID-19 vaccine”
Joint Committee on Vaccination and Immunisation^10^	“…can be vaccinated with a suitable product for their age and clinical risk group”	“…available data do not indicate any harm to pregnancy” “…should be offered vaccination at the same time as non-pregnant women, based on their age and clinical risk group”	“…no known risk associated with giving non-live vaccines whilst breastfeeding” “…may be offered any suitable COVID-19 vaccine” “…should be informed about the absence of full safety data for the vaccine in breastfeeding”
Ministry of Health, Israel^18^	“…recommended that women who are planning a pregnancy or who are undergoing fertility treatments complete the two vaccine doses before the beginning of the pregnancy”	“…recommend vaccinating pregnant women who are considered as being at high-risk” “It is our recommendation to vaccinate all pregnant women in their second or third trimester”	“It is recommended to vaccinate breastfeeding women”
Public Health England^19^	“…no need to avoid pregnancy after COVID-19 vaccination”	“…pregnant women should be offered COVID-19 vaccines at the same time as people of the same age or risk group”	“…not thought to be a risk to the breastfeeding infant, and the benefits of breastfeeding are well known”
National Advisory Committee on Immunisation, Canada^11^	NR	“…preferentially recommends that a complete vaccine series with an mRNA COVID19 vaccine should be offered”	“…individuals should continue to breastfeed after vaccination”
Society of Obstetricians and Gynaecologists of Canada^6^	“……should not be counselled to terminate pregnancy based on having received the vaccine” “…not known whether an individual should delay pregnancy following receipt of the vaccine and a risk–benefit discussion for those planning pregnancy should occur”	“Women who are pregnant or breastfeeding should be offered vaccination at any time during pregnancy if they are eligible”	“Women who are pregnant or breastfeeding should be offered vaccination at any time during pregnancy if they are eligible”

NR: not reported.

Note: These recommendations are those published up to 16 June 2021. These recommendations may have changed since preparation of this article. Notably, the Australian Technical Advisory Group on Immunisation and Royal Australian and New Zealand College of Obstetricians and Gynaecologists recommendations were changed on 9 June 2021 (the updated statement is shown in Box 1).

## Contributing factors

In the rapidly changing environment of the COVID-19 pandemic, there are challenges to effective communication of concise and consistent messages relating to COVID-19 vaccine recommendations. For women who are planning pregnancy or who are currently pregnant or breastfeeding there are issues which make deciding about vaccination especially challenging.

A recurrent issue highlighted in recommendations on COVID-19 vaccination in pregnancy is the lack of clinical trial data to inform the advice. The exclusion of pregnant and lactating women from clinical trials contributes to the lack of data. The problem is not restricted to COVID-19 vaccine trials but also affects trials of COVID-19 treatments.^23^ Pregnant women are usually excluded from clinical trials due to concerns about the physiological changes in pregnancy, potential harm to the fetus resulting from novel interventions and fear of legal liability resulting from unforeseen harm to the mother or fetus.^24^^,^^25^ Exclusion of pregnant women from trials is justified in some cases where there is a plausible belief that interventions could cause harm. Such a cautious approach, however, is often the default when participants are being recruited for clinical trials, even when there is no reason to expect that the intervention would be harmful during pregnancy.^26^ This lack of data obliges health professionals to make recommendations without clear safety and efficacy data from trials. In the context of a pandemic, new treatments and vaccines intended to protect pregnant women and their babies are often first used in this population outside the rigorous monitoring of a clinical trial. This means that only observational data are available to guide future care recommendations. More recently, clinical trials have started evaluating the safety and efficacy of COVID-19 vaccines in pregnant women. These include studies using the Moderna mRNA COVID-19 vaccine (clinicalTrials.gov identifier: NCT04958304) and observational studies following any vaccine approved by the United States Food and Drug Administration (NCT04826640 and NCT04705116).

In the absence of data from clinical trials, health professionals must seek advice from peak medical organizations (national medical colleges for obstetricians and gynaecologists) to guide their clinical management. The concerns that make researchers reluctant to include pregnant women in clinical trials are mirrored in the cautious language used in position statements issued by professional organizations, immunization advisory groups and government bodies.

## Role of medical organizations

Recommendations about COVID-19 vaccination have been issued not only by peak medical organizations but also by many national immunization technical advisory groups,^10^^,^^11^^,^^16^ peak infectious disease bodies^13^^,^^15^ and governments.^18^^,^^19^^,^^27^ National vaccine recommendations made by expert advisory groups on immunization often involve a complex and resource-demanding evaluation of the available evidence. COVID-19 has brought into focus the role of many advisory groups in advising governments about the choices of products and priority populations for vaccination and the challenges of local implementation. The growing volume of global data on vaccines contrasts with the often-limited availability of local data. Collaboration between advisory groups is therefore more important than ever.

Previous studies have attempted to understand the barriers to collaboration among national immunization technical advisory groups across countries. A cross-sectional survey of 30 European countries in 2014 identified structural concerns (such as differences in the structure of the advisory groups or the health-care systems) as one of the main barriers limiting the opportunities for collaboration.^28^ Importantly however, in 25 of the 27 countries that participated in the survey, respondents thought that there was potential for collaboration or resource-sharing among these advisory groups to support the process of developing vaccination recommendations in individual countries.^28^ In 14 of these countries respondents identified the potential benefits of sharing their evidence reviews of vaccine safety. Despite recognition of the benefits, collaboration across advisory groups remains rare. With resources stretched globally during the COVID-19 pandemic, revisiting the benefits of greater collaboration is needed.

While national immunization technical advisory groups can provide advice on implementation, their role is usually separate and independent of the implementers of vaccine programmes. Furthermore, these advisory groups provide advice on all types of vaccines, across all age groups. They may not therefore be recognized for their medical expertise among specific patient populations, such as pregnant women. Collaborating with peak medical organizations specific to the population of interest could therefore be valuable. Evidence-based decision-making processes to reach a consensus with peak medical organizations will assist in minimizing confusion and building public trust. Achieving high vaccine coverage among pregnant women is often challenging^29^ and new approaches need to be considered. One such innovation is bringing together not only national immunization technical advisory groups but also experts in the population of interest: pregnant women. The aim is to reach a consensus position and, within this, to simplify the messaging so the recommendations are clear and consistent.

## Importance of language

The statements put forth by these authorities are designed to help pregnant women and health professionals weigh the risks and benefits of vaccination during pregnancy or breastfeeding.^30^ A factor that can hamper a patient’s willingness to accept a vaccine during pregnancy, or the provider’s willingness to recommend the product, is when overly precautionary language is used. The effect of such language has been demonstrated with influenza vaccination in pregnancy. A survey administered to maternal health-care providers from 49 countries in all six World Health Organization regions found that negatively framed product information had an impact on perceived safety compared with positively framed statements.^31^ Regulatory agencies also have a role to play in maintaining consumer confidence about vaccine safety for pregnant women.^32^ When national immunization technical advisory groups and regulatory agencies work together, they can harmonize messages based on the collective safety data in pregnancy rather than individual product data.^32^

Inconsistencies in the language used in recommendations about COVID-19 vaccination in pregnant women can add to the confusion. In Table 1 we summarize a selection of the recommendations from national immunization technical advisory groups and peak medical obstetric and gynaecological organizations from around the world. This list is not exhaustive but demonstrates that, while key messages in statements from different organizations are largely similar, slightly different wording is used to convey these messages. There are also notable differences in which groups to prioritize, the timing of vaccinations and which vaccine brands to use. 

Some statements used more affirmative language which supports vaccination among pregnant women. Other statements took a more cautious, passive approach suggesting that vaccines are not recommended but not contraindicated. Although pregnant women should be well informed about the lack of evidence to support the safety and efficacy of vaccination in pregnancy, the discrepancies between these statements could make women more hesitant about receiving vaccines.

Variations in the way recommendations are worded may reflect the intention behind the recommendation. Most authorities used neutral language such as “can receive,” with some authorities emphasizing that the decision to vaccinate should be based on an individual risk–benefit assessment. Such advice places the decision-making responsibility in the hands of pregnant women themselves, supported by their health-care providers. Few authorities provided more directly positive advice, such as “should be offered”^10^ or negative advice, such as vaccination “is not routinely recommended.”^7^^,^^16^ The New Zealand Immunisation Advisory Centre recommended vaccinating high-risk pregnant and lactating women and delaying vaccination for low-risk women until after birth.^17^ More recently, pregnant women have been prioritized for vaccination in some locations, including most states in the USA and in Canada.

Safety of vaccination is often cited as the most important factor contributing to a woman’s decision whether to access a vaccine while pregnant or breastfeeding. Therefore, to build confidence in vaccination during pregnancy, advisory bodies need to address safety concerns. Guidelines for pregnant women frequently acknowledged the lack of specific safety data in this population. Only one guideline specifically acknowledged the lack of safety concerns generally, in relation to inactivated vaccines in pregnancy.^8^ Most guidelines did not address the optimal timing of vaccination during pregnancy, while others provided varying advice, ranging from “any time”^6^ to “second or third trimester.”^24^

Advice for women planning pregnancy was generally consistent between authorities, with no specific precautions advised. An exception was the European Society of Human Reproduction and Embryology who recommended: “In the absence of information on the effect of the COVID-19 vaccine on oocytes and sperm, embryo implantation and early stages of pregnancy, and to allow time for antibody development, a more cautious approach could be considered (i.e. postpone the start of antiretroviral treatment for up to 2 months).”^14^ Notably, professional societies were more likely than government agencies to provide reassurance that there is no reason to suspect COVID-19 vaccines will have an impact on fertility.^8^^,^^9^ Many guidelines specifically stated that there was no need to avoid pregnancy before or after vaccination. However, the Society of Obstetricians and Gynaecologists of Canada advised that “it is not known whether an individual should delay pregnancy following receipt of the vaccine and a risk–benefit discussion for those planning pregnancies should occur.”^6^

## Strengthening confidence

New evidence about the safety of COVID-19 vaccines is constantly emerging. Collaboration among national immunization technical advisory groups should therefore continue to be encouraged during the pandemic. This collaboration also offers the opportunity to support individual countries that may be facing new challenges due to the pandemic. For many low- and middle-income countries, the national immunization technical advisory group interprets WHO vaccine advice and other data to determine the policy recommendations best suited to their national context. An example of this is in India, where local operational guidelines were released on 2 July 2021 in response to the emerging outbreak of the Delta variant of SARS-CoV-2 in the country.^33^ This guideline cited the WHO guidance for COVID-19 vaccination of pregnant women. The guideline also referenced multiple high-income countries with existing guidelines recommending vaccination of pregnant women. The operational guideline released in India highlights the importance of consistent messaging both between and within countries.

After the evidence has been evaluated, the process of developing local recommendations is likely to be country-specific and this is where collaboration with peak medical organizations within a country may be beneficial. One solution to this challenge is to promote a collaborative approach among in-country national immunization technical advisory groups, peak medical organizations and governments. The aim will be to use consistent language across guidelines or put out a joint position statement. The Royal Australian and New Zealand College of Obstetricians and Gynaecologists and the Australian Technical Advisory Group on Immunisation have done exactly this. Before 9 June 2021, both organizations had produced their own advice (Table 1). However, with increasing evidence confirming pregnant women are at risk of more severe disease from COVID-19 and with a favourable safety profile emerging from post-vaccine licensing surveillance (predominantly from the USA),^34^ the two organizations came together to formulate an updated joint position (Box 1). The process involved reviewing the data; developing the proposed wording; sharing the proposed wording between the two organizations; incorporating suggested changes to the wording by members of organizations; and, ultimately, endorsement of the joint statement by both the advisory group and the professional organization. No challenges were experienced in reaching this consensus. The joint statement was then forwarded for approval to the Australian Ministry of Health and was published on 9 June 2021.^35^ The statement was developed for clinicians, so that obstetricians, midwives and primary-care physicians could have clear recommendations to refer to when advising pregnant women. The statement was also intended for pregnant women themselves, so it was written in a way that was clear and easily understood. The College published this statement on their website, where additional, more detailed information was available for clinicians in relation to COVID-19 vaccines and pregnancy.

Box 1 **Updated joint statement about COVID-19 vaccination for pregnant women in Australia and New Zealand**

“Royal Australian and New Zealand College of Obstetricians and Gynaecologists and Australian Technical Advisory Group on Immunisation recommend that pregnant women are routinely offered Pfizer/BioNTech COMIRNATY® vaccine at any stage of pregnancy. This is because the risk of severe outcomes from COVID-19 is significantly higher for pregnant women and their unborn babies.

Global surveillance data from large numbers of pregnant women have not identified any significant safety concerns with mRNA COVID-19 vaccines given at any stage of pregnancy. Furthermore, there is also evidence of antibody in cord blood and breastmilk, which may offer protection to infants through passive immunity.

Pregnant women are encouraged to discuss the decision in relation to timing of vaccination with their health professional.

Women who are trying to become pregnant do not need to delay vaccination or avoid becoming pregnant after vaccination.”

COVID-19: coronavirus disease 2019.Source: Department of Health of Australia, 2021.^35^

The Australian Technical Advisory Group on Immunisation then updated their decision aid for pregnant women, also publicly available to support clinicians in implementation of this policy. Significantly, this joint statement was also developed through collaboration with the New Zealand Ministry of Health and the Immunisation Advisory Centre, New Zealand, with almost identical statements on the safety of vaccination in pregnancy and breastfeeding being issued in Australia and New Zealand on the same day. The concurrent release of unambiguous, consistent messaging by three key organizations (Australian Technical Advisory Group on Immunisation, Royal Australian and New Zealand College of Obstetricians and Gynaecologists, and New Zealand Ministry of Health) is a positive step forward in this pandemic. Whether this action will increase vaccine confidence among pregnant women and increase uptake among women planning pregnancy or those breastfeeding is yet to be seen. Vaccine hesitancy remains a significant barrier to the success of the global vaccination programme, with high rates of hesitancy reported in many countries in the Western Pacific Region. Reassurance about vaccine safety for pregnant and lactating women will potentially improve confidence within the broader community within Australia and New Zealand and in other countries in the Region.

## Conclusion

Trying to keep up with the evolving evidence and changing guidelines during the COVID-19 pandemic is challenging not only for the general population but also for health professionals. National advisory groups for immunization, professional obstetric organizations and government bodies should be encouraged to coordinate their statements on COVID-19 vaccination for pregnant and lactating women and to use similar language and phrasing for greater clarity. To go a step further, authority groups should work together to develop a joint national statement to limit the variability of recommendations and avoid the need for interpretation of statements from different authoritative bodies.
